# Evaluation of Social Media Use by Emergency Medicine Residents and Faculty

**DOI:** 10.5811/westjem.2015.7.26128

**Published:** 2015-10-20

**Authors:** David Pearson, Michael C. Bond, Jason Kegg, Tyson Pillow, Laura Hopson, Robert Cooney, Manish Garg, Jay Khadpe, Michael Runyon, Leigh Patterson

**Affiliations:** * Carolinas Medical Center, Department of Emergency Medicine, Charlotte, North Carolina; † University of Maryland School of Medicine, Department of Emergency Medicine, Baltimore, Maryland; ‡ Southern Illinois University School of Medicine, Department of Emergency Medicine, Springfield, Illinois; § Baylor College of Medicine, Department of Emergency Medicine, Houston, Texas; ¶ University of Michigan Health System, Department of Emergency Medicine, Ann Arbor, Michigan; || Geisinger Medical Center, Department of Emergency Medicine, Danville, Pennsylvania; # Temple University School of Medicine, Department of Emergency Medicine, Philadelphia, Pennsylvania; ** SUNY Downstate Medical Center, Department of Emergency Medicine, Brooklyn, New York; †† Brody School of Medicine, Department of Emergency Medicine, Greenville, North Carolina

## Abstract

**Introduction:**

Clinicians and residency programs are increasing their use of social media (SM) websites for educational and promotional uses, yet little is known about the use of these sites by residents and faculty. The objective of the study is to assess patterns of SM use for personal and professional purposes among emergency medicine (EM) residents and faculty.

**Methods:**

In this multi-site study, an 18-question survey was sent by e-mail to the residents and faculty in 14 EM programs and to the Council of Emergency Medicine Residency Directors (CORD) listserv via the online tool SurveyMonkey™. We compiled descriptive statistics, including assessment with the chi-square test or Fisher’s exact test. StatsDirect software (v 2.8.0, StatsDirect, Cheshire, UK) was used for all analyses.

**Results:**

We received 1,314 responses: 63% of respondents were male, 40% were <30 years of age, 39% were between the ages 31 and 40, and 21% were older than 40. The study group consisted of 772 residents and 542 faculty members (15% were program directors, 21% were assistant or associate PDs, 45% were core faculty, and 19% held other faculty positions. Forty-four percent of respondents completed residency more than 10 years ago. Residents used SM markedly more than faculty for social interactions with family and friends (83% vs 65% [p<0.0001]), entertainment (61% vs 47% [p<0.0001]), and videos (42% vs 23% [p=0.0006]). Residents used Facebook™ and YouTube™ more often than faculty (86% vs 67% [p<0.001]; 53% vs 46% [p=0.01]), whereas residents used Twitter™ (19% vs 26% [p=0.005]) and LinkedIn™ (15% vs 32% [p<0.0001]) less than faculty. Overall, residents used SM sites more than faculty, notably in daily use (30% vs 24% [p<0.001]). For professional use, residents were most interested in its use for open positions/hiring (30% vs 18% [p<0.0001]) and videos (33% vs 26% [p=0.005]) and less interested than faculty with award postings (22% vs 33% [p<0.0001]) or publications (30% vs 38% [p=0.0007]).

**Conclusion:**

EM residents and faculty have different patterns and interests in the personal and professional uses of social media. Awareness of these utilization patterns could benefit future educational endeavors.

## INTRODUCTION

The term social media (SM) describes interactive digital platforms that are used to share information and ideas. Emergency medicine (EM) practitioners and educators use SM as tools to share medical education and healthcare applications.^1^–^5^

Residency programs are using SM increasingly for recruiting, communication, and education.^1^–^6^ Many programs report higher learner satisfaction, improved peer collaboration, increased communication, and benefits of asynchronous learning opportunities.^7^–^17^ This growing integration of SM into medical education has led some to believe that SM constitute the cornerstone platform for the future of medical education (http://bit.ly/NxV0RJ).

Despite their potential benefits, SM pose substantial potential legal, ethical, personal, and professional risks.^18^–^24^ Disclosures of private health information and breaches of professionalism issues leading to termination have been reported.^2^–^20^;^25^–^27^ In recognition of these potential risks, many hospitals and institutions have instituted policies around posting content that could have professional ramifications. In addition, residency programs have been advised to provide education regarding SM use.^28^

Despite the potential benefits and risks of SM use in EM graduate medical education, little is known about the personal and professional usage patterns of residents and the faculty of residency programs. Understanding how these physicians use SM might enhance how education is delivered and could help optimize SM use in graduate medical education. Therefore, we undertook a study that compared the personal and professional use of various SM applications by residents and faculty in EM residencies in the United States. We hypothesized that residents use SM for personal intent more than faculty members and that faculty members would be more likely to use SM for professional purposes.

## METHODS

This multi-site study was based on a voluntary, anonymous 18-question survey distributed by email in May 2013 via the online tool SurveyMonkey™. The recipients were residents and faculty whose contact information was available in the Council of Emergency Medicine Residency Directors (CORD) listserv. The members of CORD, a national EM education organization, are leaders in allopathic and osteopathic EM residencies. This study was administered by CORD’s Social Media Task Force, consisting of 14 geographically diverse educational leaders, each associated with an accredited EM residency program. The study protocol was approved by the institutional review board of the Carolinas Health Care System.

We included residents in the survey if they were enrolled in one of the 14 EM programs with a leader on the CORD SM Task Force. The number of contacts at the 14 institutions totaled 432 residents. Our goal was a 70% response rate (approximately 302 resident respondents). Additionally, residents outside this core group of 14 institutions were included if a faculty member from another institution forwarded the survey to them; the proportion from each resident group is unknown. We sent follow-up emails two and four weeks after the initial survey distribution in an attempt to increase the participation rate.

The faculty component of the study consisted of residency program directors (PDs), assistant and associate program directors (APDs), core faculty members, and others with access to the CORD listserv. A link to the survey was sent to these faculty members via the CORD listserv. We also sent follow-up emails at two weeks and four weeks in an attempt to increase the participation rate.

Sample survey questions are presented in Appendix. Specific measures included the use of SM by residents, knowledge of institutional policies regarding SM, and a comparison of SM use by residents and faculty members.

The data are summarized as counts and percentages. Between-group comparisons were performed with the chi-square or Fisher’s exact test. The analysis was performed using StatsDirect Version 2.8.0 (StatsDirect Ltd).

## RESULTS

We received 1,314 responses. The participants’ demographics are summarized in Table 1. The faculty respondents’ geographic distribution was as follows: Northeast, 32%; South, 31%; Midwest, 27%; and West, 8%. The residents had a similar geographic distribution: Northeast, 33%; South, 33%; Midwest, 24%; and West, 8% (p<0.58).

Residents used social networking sites more frequently for personal use than did faculty members. The highest frequencies of use were associated with “multiple times per day” and “daily” (Table 2). For overall personal use, 12.3% of the combined group of residents and faculty stated that they don’t use any social networking sites and 11.5% of the group reported that they use networking sites “infrequently enough to forget my password.” The barriers most frequently cited were privacy concerns (84.1%), professional boundary concerns (72.2%), lack of time (51.4%), and sites being blocked (32.7%).

Residents reported using SM markedly more than faculty for social interaction with family and friends, entertainment, and videos (Table 3). Residents used Facebook™ and YouTube™ more often than faculty, whereas faculty members used Twitter™ and LinkedIn™ more often than the residents. Overall, residents use SM sites more than faculty, notably in the daily use category (Table 4).

We then assessed interest in the use of SM for professional purposes. After combining the resident and faculty groups, we found that 28.7% had a “very high” or “high” level of interest, 30% were neutral in their interest, and 41.3% had a “low” or “very low” interest. Residents and faculty members had similar levels of “very high” or “high” interest (28% vs 30%) and “low” or “very low” interest (39% vs 44.6%) for the use of SM in a residency environment (Table 5). Residents were most interested in professional SM use for open positions/hiring (30% vs 18% [p<0.0001]) and videos (33% vs 26% [p=0.005]) and were less interested than faculty with award postings (22% vs 33% [p<0.0001]) and publications (30% vs 38% [p=0.0007]) (Table 6).

One fourth of the respondents said their program has an official SM policy in place, and 15% reported they did not have such a policy. Eighteen percent reported being covered under hospital, corporate, or institutional policy, and 37% did not know if a policy had been enacted. Less than half (40.3%) of the respondents said their residency programs had a SM page/site, and 28.8% of respondents were not sure about the existence of a site. Of those reporting a SM page/site, 30% said the site manager or administrator was a resident, 27% reported that this role was filled by the PD or an APD, 19% said that a faculty member other than the PD or an APD administered the site, and 14.7% reported that program coordinator filled this responsibility.

## DISCUSSION

The results of our survey indicate that, for personal use, EM residents are more likely to use SM than are EM faculty members. The frequency of use of specific SM modalities varied between the two groups of respondents. For professional purposes, residents and faculty had highly varied levels of interest in the use of SM in a residency environment.

Given the expanding presence of SM in graduate medical education, understanding utilization patterns is essential to integrating them into educational programs. SM have the potential to facilitate didactic learning, capture feedback from learners, and enhance educational discussions, but if educators and learners are familiar with different SM tools, program developers face major challenges. We suggest that each residency program should explore its faculty and residents’ use patterns before implementing a new SM-based curriculum. For example, our survey study revealed that faculty members use Twitter™ more commonly than do residents; so, before a Twitter™-based curriculum is deployed, residents and faculty members should be educated about the site to maximize participation and satisfaction. Residents and faculty members should be made aware of institutional policies regarding the use of social media. Before launching an educational program that includes the use of SM, program administrators should talk with information technology personnel and hospital administrators to ensure appropriate access to the educational resources (e.g., Facebook™, Twitter™, and YouTube™). A third of the physicians who responded to our survey reported being blocked from sites of interest by hospital networks. The elimination of technology barriers is essential to the successful use of SM in residency education.

We were surprised that 41% of our study group expressed “low” or “very low” interest in using SM for professional purposes. The highest levels of interest in this category were associated with obtaining information about the residency program, viewing articles for discussion during Journal Club, and retrieving publications (Table 6). This information could provide a starting point from which to launch programs based on SM in a residency program. Additionally, SM can be used to address Milestones 15, 18, 19, 20, and 21, which cover medical knowledge, technology, practice-based performance improvement, professional values, and accountability, respectively.^29^–^30^

## LIMITATIONS

Limitations of this study include the unavoidable limitations inherent to the collection of self-reported information via a survey. Our initial intent was to reach out to only 14 residencies; the study group expanded beyond that focus when the survey was distributed more broadly by faculty members on the CORD listserv. Thus, we received more responses than we anticipated (772 instead of 302), and we were not able to tally the number of programs and residents that actually received the survey (i.e., our response rate is unknown). The faculty response rate is also unknown, because the total number of individuals on the CORD listserv is unknown and our emails could have been forwarded to faculty not on the listserv. CORD membership includes nearly all EM residency PDs and APDs; therefore, 542 faculty responses represents a large proportion of residency leaders. Finally, respondents could have responded more than once, as the survey was anonymous.

## CONCLUSION

Emergency medicine residents and faculty members are different in their patterns regarding the use of social media for personal purposes and in their interest in using social media for professional purposes. Awareness of these varied utilization patterns may benefit future educational endeavors.

## Figures and Tables

**Table 1:** Demographics.

Total responses	1,314
Residents	772 (59%)
Faculty	542 (41%)
Program directors	81/542 (15%)
Assistant or associate program directors	114/542 (21%)
Core faculty	244/542 (45%)
Other faculty	103 (19%)
Sex: Male	828 (63%)
Age <30 years	526 (40%)
Age 31–40 years	512 (39%)
Age >40 years	276 (21%)
Faculty completed residency >10 years ago	578 (44%)

**Table 2:** Frequency of personal use of social media networking sites by faculty and residents.

How often do you use social networking sites?	Residents (n=742)*	Faculty (n=496)*	p
Daily	221 (30%)	118 (24%)	0.001
Infrequently enough to forget my password	49 (7%)	94 (19%)	
Monthly	42 (6%)	32 (6%)	
Multiple times a day	231 (31%)	112 (23%)	
Several times a week	129 (17%)	69 (14%)	
Weekly	70 (9%)	71 (14%)	

* 46 faculty members and 30 residents did not answer this question.

**Table 3:** Reasons for personal use of social media.

	Residents (n=772)	Faculty (n=542)	p
News	334 (43.3%)	218 (40.2%)*	0.27
Entertainment	469 (60.8)	256 (47.2)	<0.0001
Videos	321 (41.6)	175 (23.3)	0.0006
Research	121 (15.7)	71 (13.1)	0.19
Events	220 (28.5)	120 (22.1)	0.01
Networking	370 (47.9)	225 (41.5)	0.02
Social (family/friends)	643 (83.3)	351 (64.8)	<0.0001

**Table 4:** Use of specific social media sites.

	Residents (n=772)	Faculty (n=542)	p
Facebook™	661 (85.6%)	364 (67.2%)	<0.0001
Twitter™	147 (19.0)	138 (25.5)	0.005
LinkedIn™	119 (15.4)	173 (31.9)	<0.0001
YouTube™	408 (52.8)	248 (45.8)	0.01
Ning™	1 (0.1)	2 (0.4)	0.57
Blogs	166 (21.5)	120 (22.1)	0.78

**Table 5:** Level of interest in using social media in residency environment.

	Residents (n=762)	Faculty (n=529)	p
Very high	87 (11.4%)	58 (11.0%)	<0.001
High	124 (16.3%)	102 (19.3%)	
Neutral	254 (33.3%)	133 (25.1%)	
Low	154 (20.2%)	85 (16.1%)	
Very low	143 (18.8%)	151 (28.5%)	

**Table 6:** Information of interest to residents and faculty when using social media.

	Residents (n=772)	Faculty (n=542)	p
Current providers	108 (14.0%)	96 (17.7%)	0.07
New providers	103 (13.3)	83 (15.3)	0.31
Open positions/hiring	231 (29.9)	99 (18.3)	<0.0001
Residency program	416 (53.9)	302 (55.7)	0.51
Services, departmental	103 (13.3)	88 (16.2)	0.14
Awards	172 (22.3)	176 (32.5)	<0.0001
Publications	235 (30.4)	204 (37.6)	0.0007
Research	220 (28.5)	180 (33.2)	0.07
Articles/journal club	330 (42.7)	226 (41.7)	0.7
Videos	255 (33.0)	140 (25.8)	0.005
