# Supplementary material for: Evaluation of Social Media Use by Emergency Medicine Residents and Faculty
Source: West J Emerg Med. 2015 Oct 20;16(5):715–20. doi: 10.5811/westjem.2015.7.26128 (PMC4644040; doi:10.5811/westjem.2015.7.26128)
Supplement: Supplementary file 1 [file wjem-16-715-s001.pdf]

Appendix A. Questions from the Social Media Task Force Survey

What social networking sites do you currently use? Indicate all that apply

- Facebook
- Twitter
- LinkedIn
- YouTube
- Ning
- Blogs
- Others not listed above
- None (I don’t use social networking sites)

How do you utilize social networking sites for personal use? Indicate all that apply.

- News
- Entertainment
- Videos
- Research
- Events
- Networking (colleagues/peers)
- Social (family/friends)
- I don’t use social networking sites

How often do you utilize social networking sites?

- Multiple times a day
- Daily
- Several times a week
- Weekly
- Monthly
- Infrequently enough to forget my password

What is your level of interest in utilizing social media in the residency environment?

- Very high
- High
- Neutral
- Low
- Very low

Does your residency have an official social media policy?

- Yes
- No
- In Process
- Covered under Hospital, Corporate, or Institutional policy
- Don’t know

What barriers have you encountered in using social media? (Indicate all that apply)

- Privacy Concerns
- Professional Boundary Concerns
- Sites Are Blocked
- Corporate Barriers
- Lack of Time/Too Busy
- Complicated/Confusing to Use

Please select the following items you would likely be interested in using social media to promote.

Indicate all that apply.

- Current Providers
- New Providers
- Open Positions/Hiring
- Residency Program
- Services – departmental services
- Awards
- Events
- Publications
- Research
- Articles / Journal Club
- Videos
- Other (with free text space to describe

Does your residency have a social media page/site?

- Yes
- No
- Not sure

If your residency has a social media page/site, who manages or acts as the administrator over the page/site?

- Program Director
- Associate or Assistant Program Director
- Faculty member (not PD or APD)
- Program coordinator
- Non-EM Med Ed Coordinators/Administration
- Resident
- Other (Please describe)

Please rate the following based on the scale: Strongly disagree, disagree, neutral, agree, and strongly agree.

- There are no issues with a faculty member and a resident being Facebook “friends.”
- There are no issues with a health care provider being Facebook “friends” with a patient if the patient offers.
- Social media interactions between faculty and residents have the potential to compromise evaluations during residency training.
- Social media interactions between faculty and residents have the potential for educational benefit.
- Social media interactions between health care providers and patients have the potential to compromise privacy.
- Postings on social media should be considered in resident evaluations of professionalism.
- Postings on social media should be considered during evaluation of residency applicants.
